# The Implementation of Measurement-Based Care in the Context of Telemedicine: Qualitative Study

**DOI:** 10.2196/41601

**Published:** 2022-11-24

**Authors:** Jen Van Tiem, Elizabeth Wirtz, Natalie Suiter, Amanda Heeren, Lindsey Fuhrmeister, John Fortney, Heather Reisinger, Carolyn Turvey

**Affiliations:** 1 Department of Veterans Affairs, Health Services Research & Development Center for Access and Delivery Research and Evaluation Iowa City Veterans Affairs Health Care System Iowa City, IA United States; 2 Department of Veterans Affairs Office of Rural Health Veterans Rural Health Resource Center-Iowa City Iowa City VA Healthcare System Iowa City, IA United States; 3 Department of Psychiatry Carver College of Medicine University of Iowa Iowa City, IA United States; 4 Department of Veterans Affairs, Health Services Research & Development Center of Innovation for Veteran-Centered and Values-Driven Care Veterans Affairs Puget Sound Health Care System Seattle, WA United States; 5 Department of Psychiatry and Behavioral Sciences University of Washington School of Medicine Seattle, WA United States; 6 Institute for Clinical and Translational Science University of Iowa Iowa City, IA United States; 7 Department of Internal Medicine Carver College of Medicine University of Iowa Iowa City, IA United States

**Keywords:** telehealth, patient-reported outcome measures, measurement-based care, health information technology, data visualization

## Abstract

**Background:**

The Measurement Based Care in Mental Health Initiative launched by the Department of Veterans Affairs in 2016 is an example of an evidence-based practice that uses patient-reported outcome measures (PROMs) to improve patient outcomes. The acceptance of measurement-based care (MBC) among Veterans Affairs providers is relatively high. However, there are barriers to MBC for telehealth providers. Health information technologies might afford opportunities to address some of the barriers related to the uptake of MBC.

**Objective:**

This paper reports on an implementation effort to integrate MBC into mental health care telehealth practice using eHealth solutions.

**Methods:**

Qualitative data were generated from 22 semistructured interviews with psychiatrists (n=4), psychologists (n=3), social workers (n=3), nurses (n=6), a pharmacist (n=1), and administrative staff (n=5) who provide telemental health care through a community-based outpatient clinic in the rural Midwestern United States. The interviews were conducted during the pilot phase of an implementation initiative to increase the adoption of MBC by revising clinic workflows to integrate the use of eHealth technologies. Data were analyzed using thematic analysis.

**Results:**

Time burden and workflow issues were the most common barrier to provider adoption of MBC; sharing and reviewing pencil-and-paper measures and results in the same room was no longer possible in novel telehealth workflows necessitated by the COVID-19 pandemic. Providers voiced concerns about how long it would take to collect, adequately score, interpret, share, and document the PROMs during the telehealth visit. Concerns about time might also correspond to a gap in providers’ familiarity with these assessments, greater comfort in assessing symptoms through clinical interviews, and being accustomed to using the assessments as screening tools more so than longitudinal outcome measures. Capacities associated with eHealth technologies may address workflow concerns and promote providers’ understanding and use of the measures as tracking tools.

**Conclusions:**

The need to use limited appointment time well was a top priority for telemental health providers. eHealth technologies provided operative supports that protect time in appointments by shifting when and how PROMs are collected. Bolstering providers’ familiarity with how to use PROMs in the course of treatment may impact providers’ buy-in by encouraging them to reconsider how sharing and acting on PROMs could be time well spent.

## Introduction

The Measurement Based Care in Mental Health (MBC in MH) Initiative launched by the Department of Veterans Affairs (VA) in 2016 is an example of an evidence-based practice that uses patient-reported outcome measures (PROMs) to improve patient outcomes [1-3]. In measurement-based care (MBC) systems, patients complete standardized assessments such as the 9-Item Patient Health Questionnaire at regular intervals, and providers and patients use the results of these assessments to help them understand symptom presentation, identify targets for intervention, and monitor progress toward treatment goals [4]. By reviewing trends over time, providers and patients discern positive or negative changes to inform clinical decisions and psychotherapy approaches [4-6]. The VA MBC in MH Initiative highlights 3 essential components through a campaign using the slogan “Collect, Share, Act” [7].

VA providers are agreeable to MBC, but use is low and varies by discipline [8]. Early documented barriers ranged from an objective lack of resources (eg, protected time) to subjective concerns about the utility of the assessments relative to direct clinical interview [9]. Low provider use of MBC persists [10,11], even as evidence about the positive impact on outcomes supports the use of MBC [6,12]. Research has consistently documented that patients generally appreciate MBC, especially how it promotes shared decision-making [13-16]. The use of MBC dropped among VA providers at the start of the COVID-19 pandemic [17], presumably because of the increase in telemental health [18], although the full impact on the delivery of clinical care, for both patients and providers, is still being understood [19-21].

Health information technologies might address some of the barriers related to the uptake of MBC especially in the context of telehealth [22,23]. Prior to the COVID-19 pandemic, there was a call to better understand “remote measurement-based care (RMBC)” [24], that is, obtaining measures independent of appointment time using patient-facing electronic platforms to deliver assessments. Following the expansion of telemental health during the COVID-19 pandemic, researchers have just begun to publish findings about the integration of MBC into telemental health [20,25]. More research needs to be done, especially given the potential of eHealth technologies to address persistent time burden and workflow barriers by automating the asynchronous administration of PROMs. As well, there is very little information on the perceived utility or functionality of the visualizations that these technologies can produce to show a graphical representation of a patient’s reported symptoms over time [24,26].

The VA is promoting the use of electronic capture of PROMs via eHealth solutions. Using these technologies, providers can administer PROMs electronically. The results are immediately scored and available for integration into the medical record without requiring synchronous administration or data entry by providers. This paper reports on an implementation effort to integrate RMBC into clinical telehealth practice using eHealth solutions. Our analysis explores the full meaning of providers’ perceptions of time and workflow barriers and makes a novel finding about how the visualizations that the eHealth applications produce offer providers the opportunity to develop a narrative of treatment.

## Methods

### MBC in VA

The MBC in MH Initiative in VA launched in 2016; the goal of the initiative was to rapidly implement MBC and position MBC as the new standard of care [2]. To achieve this goal, members of the initiative rolled out MBC to 176 general and specialty care mental health programs, as well as Primary Care Mental Health Integration programs [2]. Researchers and implementation scientists involved in the implementation effort have published on the barriers and facilitators, as well as lessons learned during implementation [2,8,10,27]. They identified a lack of appropriate “technology” as a barrier to the implementation of MBC, although they suggested that “provider attitudes” and “organizational climate” also likely served as barriers [2].

The software platforms that were available at the VA in 2016 (Mental Health Assistant, VA Office of Information and Technology; and Behavioral Health Lab, Capital Solution Design) lacked both patient- and provider-facing features that would have facilitated a robust MBC practice [2]. Both Mental Health Assistant and Behavioral Health Lab were provider-facing software platforms, meaning that providers had to collect the data from patients, usually through direct interview or asking patients to complete pencil-and-paper forms. Providers or administrative assistants then had to enter the data into the medical record manually. Although some clinics were able to implement collection via a tablet in the waiting room, the predominant form of data collection was through direct assessment by the provider and hand entry into the medical record. Data from PROMs had to either be manually entered by the provider during appointments, entered with a tablet in the waiting room and then entered into the electronic medical record, or collected via pen and paper [10]. Some clinics explored using secure messaging via the patient portal (My Health*e*Vet; United States Veterans Health Administration) to share assessments between patients and providers [25], but this method did not resolve either the time burden or workload barriers. At the time, patient-facing eHealth applications that have been used in the context of RMBC [24] were not available in VA and were not part of the initial implementation of MBC.

The VA has only recently started exploring patient-facing eHealth applications such as BHL Touch (Capital Solution Design) and MH CheckUp (VA Mobile). MH CheckUp was first rolled out in December 2020 and BHL Touch was rolled out in July 2021. Our implementation effort entailed understanding how to use these eHealth applications to facilitate the workflow processes associated with MBC, including collecting, sharing, and acting on PROMs. Human/user-centered design processes structured our implementation according to 4 phases: “Discover,” “Design,” “Build,” and “Test” [28]. The purpose of our study was to develop workflows for clinicians that addressed the potential technological, organizational, and attitudinal barriers to the use of MBC. This paper reports on data generated during the “Discover” phase.

### Setting

We conducted a rapid ethnographic assessment [29] of the workflows of providers based in rural community-based outpatient clinics (CBOCs) that served as satellite clinics for VA Medical Centers often located in urban settings. Providers were familiar with the routine collection of patient-reported outcomes in the context of protocols for evidence-based practices such as cognitive processing therapy, although this was not necessarily labeled as MBC. They had all also been made aware of new eHealth technologies such as MH CheckUp and BHL Touch; however, their engagement with these applications was dependent upon their own interest. Of all of the clinicians we talked to (n=18), there were 5 people who already had some experience using these eHealth applications in the context of treatment.

This work supported the operational goals of the Office of Mental Health and Suicide Prevention and the Office of Rural Health within the VA.

### Ethical Considerations

Our project procedures were reviewed by the University of Iowa Institutional Review Board (# 202009601) and determined to be non–human subjects research. Additionally, the project was reviewed by the Iowa City VA Research and Development Committee.

### Participants

We recruited staff members (n=26) who provide telemental health services to patients at 2 CBOCs in the rural Midwestern United States. A total of 22 staff members agreed to participate; they included psychiatrists (n=4), psychologists (n=3), social workers (n=3), nurses (n=6), a pharmacist (n=1), clerical staff (n=2), program managers (n=2), and the regional telehealth point of contact (n=1). Although we only report on interview data with psychiatrists, psychologists, social workers, pharmacists, and the telehealth point of contact in this paper, it was important to speak with clerical staff, nurses, and program managers to understand the organizational climate of the CBOCs. Not only did interviews with these individuals help us ask better questions of the providers, but they will also inform the decisions we make in subsequent phases of the study, including “Design” and “Build.” Moreover, some of these supporting staff members were part of the workflow related to collecting PROMS from patients.

### Procedure

We conducted direct observations of weekly web-based team meetings and semistructured interviews over videoconferencing. The program managers on each of the teams facilitated our entry into the weekly team meetings. We started observing weekly team meetings in December 2020 and continued through 2022. We took detailed field notes of the discussion of workflows that shaped the day-to-day work of the teams. Attending weekly team meetings served four purposes: (1) it acclimated the qualitative analysts (who are both trained in anthropology and not clinicians) to the technical language that the team members used when talking about their work; (2) it helped in the development of rapport, so that it was ultimately easier to recruit participants for interviews, and during those interviews, both the participant and interviewer had a sense of shared experience; (3) it helped us notice potential workflow and organizational barriers to the implementation of the eHealth technologies; and (4) in helping us notice those potential barriers, we were able to tailor our interview probes to the context. We maintained our presence in weekly meetings even after the interviews were completed to track emerging workflow and organizational barriers as we moved into the “Design” phase of our implementation. We used this method as a workaround when our original plan to conduct site visits was derailed due to COVID-19 travel restrictions.

Our early field notes informed the development and refinement of our interview guide (Multimedia Appendix 1). Our interviews addressed staff perceptions of using PROMs in clinical care; their experiences integrating standard assessments into individual appointments, as well as treatment over time; and finally, their techniques for reviewing the assessments with their patients. We recruited staff members via email and conducted interviews from May 2021 to October 2021. Participation was voluntary and we obtained verbal consent. Interviews were recorded and transcribed; they ranged from 18 minutes to 60 minutes and averaged 35 minutes.

### Data Analysis

In all, 2 qualitative analysts conducted a thematic analysis [30] of the interview data. The analysts coded each interview together. Using inductive and deductive coding [31], we identified themes related to workflow, time, patient flow, data management, technology, job role, protocols, treatment approach, and perceptions of MBC.

## Results

### Overview

The barriers that have been documented for in-person care were also barriers to MBC in the context of telehealth. We heard providers voice concerns about how long it would take and how much coordination it would entail to adequately score, interpret, share results with patients and document the PROMs. We also found that the providers who were already engaging with eHealth applications noticed how these technologies can help resolve some of the time burden and workflow barriers in both telehealth and in-person visits.

In asking providers to reflect on their experiences with MBC, we surfaced latent barriers couched within the larger umbrellas of workflow and time. Concerns about time might correspond to providers’ training and preference in assessing symptoms through clinical interviews rather than standard assessments. These characteristics, combined with being accustomed to using the assessments as screening tools more so than longitudinal outcome measures, might contribute to providers’ hesitancy to use MBC as a tool for shared decision-making and treatment planning.

In our discussions with providers, we made a novel finding about the potential of the visualizations that the eHealth technologies can create. Researchers have noted how tracking patient’s responses over time using visualizations is an unexplored potential benefit of RMBC with eHealth technologies [24,26]. We found that the visualizations helped providers develop a narrative about the course of the patient’s illness and understand trends of symptom severity over time.

### Telehealth, MBC, and Workflow

At the time of the interviews, providers were still adjusting to changes in their workflow precipitated by the COVID-19 pandemic. The workflows providers had been using to administer PROMs were no longer possible. One psychiatrist explained how, prior to the pandemic,

Usually the patients...would check in in a kiosk and that would flag them to fill out or to receive a paper copy of a questionnaire, And then the patient [would] fill it out on paper and then someone physically on site would upload that into...our electronic medical record, and then we would get it that way. And then the pandemic hit and everyone’s doing video visits to home, which is very exciting. And now trying to catch up and find a way to capture um, that workflow, but all electronically.

Psychiatrist and telehealth point of contact, multiple CBOCs

When we spoke with providers, they had been using several telehealth formats, including phone visits and video-based visits such as video telemedicine and VA Video Connect (VA Mobile) visits. For video telemedicine visits, patients come into the CBOC and have their visit via video chat with a provider who is either in another room or at another clinic location. For VA Video Connect visits, patients stay at home and have their visit with a provider who is at a clinic or at home. Providers anticipated workflow barriers to integrating PROMs into telehealth appointments, including questions about when to distribute the PROM, how to get it back, and how to get it back in time for it to be used during the appointment. One psychiatrist described their experience in using MBC during in person visits and how “there’s a lot of pieces.” They said,

So when I think about Tele Health, there’s the phone which we’re trying to go away from, and then there’s [VA’s telehealth platform]. So some of my patients like I log in, it’s the start of the appointment we’re both there, fantastic. We’re having an appointment that’s great and I’m trying to get through everything that I need to do to actually take care of a patient. I’m trying to throw in some clinical reminders when I remember. I’m trying to schedule them for their next appointment. And then how I would actually do the measurement based care part? I’m not sure because I’m used to being able to like throw them a measure while I multitask and now I can’t...in a perfect scenario, if they show up, it’s possible that if we had a way for [our teleadministrative staff] to know, like “hey, this person needs XY and Z,” then maybe [the Veteran] could be handed that, but then they’re like trying to do it during the appointment. Do they turn it in after? Where does it go? Uhm, is there something that they could be sent ahead of time that they could do at home and it could be ready in time for the appointment?

Psychiatrist, CBOC 1

The use of electronic applications to conduct assessments prior to scheduled visits addressed many of the workflow issues that this provider described. Some providers were already making use of available eHealth technologies on an individual basis. With these technologies, providers schedule assessments in advance of appointments. Patients receive a link over email or SMS text message, and they complete the assessments; the results are shared and discussed in the upcoming mental health visit. Some applications allow the provider to sync results directly into the electronic medical record, so a patient’s responses to assessments are easily documented, integrated into the visit note, and shared with all the different providers that care for that patient within that institution. A few providers we spoke to had already started using some of the eHealth applications. One of these psychiatrists said,

When I ask Veterans, “Oh how did it go on your end?” the majority answer on their phones...So it seems like text is the best--, has had the most success or the easiest for them to respond. I like that I don’t have to do anything. I can just upload it and it’s right there.

Psychiatrist and telehealth point of contact, multiple CBOCs

A social worker reported a similar experience:

What I like about it is I can schedule as many...I can have them do more than one...like if I’m seeing somebody weekly and I want to know how they’re doing with whatever one of the assessments I choose to use. I’m able to put it on a weekly basis without having to go back in there and redo it. So that’s what I like about it and then I’m able to see it in [the electronic medical record]...so that morning...before their appointment I can see what the results were.

Social worker 1, CBOC 2

Providers who had already started using some of the eHealth solutions helped us notice how the functionality of the applications could solve some of the workflow barriers identified by providers who had not yet tried to use the eHealth technology.

### Telehealth, MBC, and Time

Confirming findings already published about the use of MBC, many providers mentioned how time burden was a potential barrier. However, we also noticed that when providers talked about time, they often talked about how they already used the clinical interview to generate the same information the assessments would capture. The relative value of MBC to clinical interviews was not clear. A psychologist described how,

It is time consuming and...it’s either, it doesn’t add as much as I would want it to the clinical interview...and I have to ask those questions anyway, um, PHQ-9 [9-item Patient Health Questionnaire] and GAD [General Anxiety Disorder], I feel like I do them and they don’t always lead to a more in-depth conversation.

Psychologist 1, CBOC 2

Providers felt that it took a long time to do the assessments because they were not sure why they were doing them, or what value the assessments added. For example, one psychiatrist talked about how,

It takes time to do it, and then once you get it, you have to then take more time to understand...what it’s actually saying. Meanwhile, you could just do like a narrative thing or subjective interview where you’re...you talk to them about their sleep and you immediately get to, ‘I'm sleeping too much’ and then you have the conversation about sleeping.

Psychiatrist 3, CBOC 1

eHealth technologies addressed this concern somewhat by moving some of this work outside of the appointment, as patients were encouraged to complete the PROMs on their own time.

### Telehealth, MBC, and Building a Narrative

Helping to resolve workflow and time barriers would not address providers’ preferences for understanding a person’s lived experience through clinical interview. On the one hand, providers voiced concern that the score on an assessment is a snapshot in time and often decontextualized from a person’s lived experience. One psychiatrist worried how,

They come in and they’re under a lot of stress because their dog died, and their scores are gonna shoot up...with time it should come, you know, back down, and I do the treatment, it will keep improving. But that’ll take time to track.

Psychiatrist 3, CBOC 1

The use of the same PROMs as both a screening tool and an MBC tool led to persistent confusion. Rather than using the assessments to shape a meaningful treatment plan and inform clinical decision-making, most providers continue to view standard assessments as screening tools rather than tools for longitudinal assessments of patient-reported treatment outcomes. Moreover, many providers lacked training about the specific content validity and psychometrics of the available measures. Another psychiatrist remembered how,

I think initially I had to like look [many of the scales] up, --not all of them, but lots of them. Like I [didn’t] know what [some were]...like are these useful scales?...So that took a lot of time and then [it] ended up being like most of them [were] not really useful for my purposes...and also I don’t think...the information [for] interpreting the results...you have to look that up separately if needed.

Psychiatrist 1, CBOC 2

Providers that used the assessments as more than screening tools talked about using the patient’s responses to shape a narrative about treatment. One social worker, adept at cognitive processing therapy for posttraumatic stress disorder, explained how the assessments help her understand how treatment was going. She described how,

I’m doing something like cognitive processing therapy, and I do a consistent uh, PCL [posttraumatic stress disorder symptom rating scale]...I expect those symptoms when we start to increase and spike, and then I expect to see them decrease and so if I don’t see that happening, if I don’t see that spike, then I-I know we’re not really touching on things that are bothering them, that we’re still really surface and if over time I don’t see a decrease, I know that we’re really not scratching what we need to hit to get them to process the information well enough.

Social worker 1, CBOC 1

eHealth technologies offer providers the ability to visualize data from the assessments to track the trajectory of a patient’s symptoms over time. The graph might be very useful for helping providers build an account of the course of treatment. One social worker reflected on their experience with visualizations provided by eHealth technologies prior to working with the VA; they remembered how,

I had one guy...it was actually pretty amazing...he was super depressed and then...you could see...right along with his mood...his chart totally changed, and he actually got to a point where [he said] “I’d rather actually pay for going to jujitsu classes than therapy. At this point, I’m feeling pretty good.”...I thought that was a cool way to be able to do it.

Social worker 2, CBOC 1

The visual functionality reinforces the assessments as tracking tools, as the graph potentially facilitates conversations about “trends over time.” Providers no longer have to analyze and create a way to present the long-term data, making it easier to visualize a patient’s progress over time. One psychiatrist who had already started using one of the eHealth technologies reflected how,

I like how quickly it shows the trend...very user-friendly...I think a big value is having the response before the start of the appointment...it’s really nice going into the appointment knowing at least on paper things look better, things look worse, than when I last saw them...So you’re already kind of thinking a little bit about what the next step might be...it kind of helps tailor [my plan] right off the bat a little.

Psychiatrist and telehealth point of contact, multiple CBOCs

Our findings indicate that not all providers were sure how to use the assessments as a method for developing an understanding of a patient’s lived experience and may have perceived the time spent doing the assessments as time not spent effectively. The above reflection, from a psychiatrist who felt successful using the PROMs to shape their clinical decisions, suggests that eHealth technologies can help providers develop a narrative of treatment that they can use to tailor their treatment plans.

## Discussion

### Principal Findings

Promoting the uptake of MBC in telehealth requires addressing the issue of time burden, which necessitates both (1) acknowledging the limited time in appointments by facilitating the administration of PROMs via SMS text messaging or email before appointments and (2) satisfying telemental health providers’ need to use time well by increasing their familiarity with how to use the assessments to measure response to treatment. eHealth technologies facilitate the administration of assessments prior to appointments and, thus, pose a pragmatic solution to concerns about time, especially in the context of telehealth. The additional functionality associated with eHealth technologies (eg, graphs that visualize patients’ responses over time) has the potential to increase providers’ awareness of assessments as tracking tools that can facilitate setting goals and following progress toward those goals rather than simply as screening tools.

### Comparison With Prior Work

Our findings confirm and extend findings in the extant literature about how time burden is a formidable barrier to the adoption of MBC [9,10]; further, our findings suggest that provider perceptions of time burden are related to unfamiliarity with PROMs as means for tracking symptoms over time. Not all mental health care providers receive training about psychometrics and the validity and reliability of standard mental health assessments. Targeted training on specific PROMs, as well as increasing awareness of the aspects of MBC, including Collect, Share, and Act, may increase provider use. Providers may reconsider the time it takes to administer (Collect) and discuss (Share and Act) as an invaluable use of time if they better understand how the information gleaned from the assessments (ie, objective measures of symptoms) can be used in concert with clinical interviews (ie, patient-lived experience) to shape treatment.

### Limitations

Although we recruited a diversity of providers from different disciplines and roles within the clinic, our sample is small and only represents 2 CBOCs, both of which were part of the VA health care system. Although our findings would be strengthened by comparison to more diverse clinic settings, our findings reflect previous studies’ findings in different settings; moreover, our qualitative methods allowed us to expand upon and clarify this previous work. Our sample size will grow as our implementation effort continues and we continue to report on our findings.

### Conclusions

The adoption of MBC into existing professional practice and the implementation of such programming into a telehealth workflow is a complex process. Promoting the uptake of MBC in telehealth requires addressing the issue of time burden, which necessitates both (1) acknowledging the limited time in appointments by facilitating the administration of PROMs before appointments and (2) satisfying telemental health providers’ need to use time well by increasing their familiarity with how to use the assessments to set treatment goals. eHealth technologies facilitate the administration of assessments prior to appointments and, thus, pose a pragmatic solution to concerns about time. The additional functionality (eg, graphs that visualize patients’ responses over time) has the potential to increase providers’ awareness of assessments as tracking tools that can facilitate setting goals and following progress toward those goals.

## Appendix

Multimedia Appendix 1 Interview guide.

## Abbreviations

CBOC: community-based outpatient clinic
MBC: measurement-based care
MBC in MH: Measurement Based Care in Mental Health
PROM: patient-reported outcome measure
RMBC: remote measurement-based care
VA: Department of Veterans Affairs
