# Supplementary material for: The Implementation of Measurement-Based Care in the Context of Telemedicine: Qualitative Study
Source: JMIR Ment Health. 2022 Nov 24;9(11):e41601. doi: 10.2196/41601 (PMC9732750; doi:10.2196/41601)
Supplement: Multimedia Appendix 1 [file mental_v9i11e41601_app1.docx]

**Appendix 1: Interview Guide**

Overall, how do you feel about using regular standard assessments in your clinical care with patients?

- Do the assessments give you information beyond what you would glean from meeting with the person?
- Do you feel using assessments impacts your flexibility in making decisions about what types of interventions to use?

From the list of screens used in BHL:

- - How do you decide which assessments to use with which patients?

How do you integrate standard assessments into your visit workflow?

- Do you do anything before the visit to prepare to discuss the assessments?
- When during the appointment do you and your patient review the results? How is this review different during phone, telemedicine, and face-to-face visits?
- Are there any action steps you need to take related to the assessments after the visit?

Do you see patients more than once? If so, how do you integrate standard assessments into the course of treatment over time?

- How do you access the previous assessments?
- How do the assessments shape point of care decision making? Can you give us an example?

***(if this hasn’t already been discussed)*** Walk us through a BHL workflow with a new patient. Help us understand what works and what does not work about the process of using the BHL tool to do assessments (i.e., logging in to BHL, doing an assessment, and making sure the information travels from BHL to MHA to CPRS).

How do you present the assessments, and the information gleaned from them, to your patients?

- How does this shape the way patients engage with you? with treatment?
- What do you imagine patients get out of this process of doing assessments and reviewing the results?

How is Measurement Based Care different from what you already do with assessments?

What does successful use of MBC look like?
